# Testosterone identifies hatchling sex for Mojave desert tortoises (*Gopherus agassizii*)

**DOI:** 10.1038/s41598-023-41677-2

**Published:** 2023-09-08

**Authors:** M. A. Walden, Kevin J. Loope, Elizabeth A. Hunter, Stephen J. Divers, Jessica R. Comolli, Todd C. Esque, Kevin T. Shoemaker

**Affiliations:** 1 Department of Natural Resources and Environmental Science, University of Nevada, Reno, Reno, NV USA; 2 Department of Fish and Wildlife Conservation, Virginia Tech, Blacksburg, VA USA; 3 Department of Biology, Georgia Southern University, Statesboro, GA USA; 4 U.S. Geological Survey, Virginia Cooperative Fish and Wildlife Research Unit, Department of Fish and Wildlife Conservation, Virginia Tech, Blacksburg, VA USA; 5 Department of Small Animal Medicine and Surgery, College of Veterinary Medicine, University of Georgia, Athens, GA USA; 6 U.S. Geological Survey, Western Ecological Research Center, Boulder City, NV USA; 7 Present Address: Department of Fisheries Biology, California State Polytechnic University, Humboldt, Arcata, CA USA; 8 Present Address: Department of Veterinary Services, Miami Seaquarium, Key Biscayne, FL USA

**Keywords:** Ecophysiology, Gonadal hormones

## Abstract

The threatened Mojave desert tortoise (*Gopherus agassizii*) exhibits temperature-dependent sex determination, and individuals appear externally sexually monomorphic until sexual maturity. A non-surgical sex identification method that is suitable for a single in situ encounter with hatchlings is essential for minimizing handling of wild animals. We tested (1) whether plasma testosterone quantified by enzyme-linked immunosorbent assay differentiated males from females in 0–3 month old captive hatchlings, and (2) whether an injection of follicle-stimulating hormone (FSH) differentially elevates testosterone in male hatchlings to aid in identifying sex. We validated sex by ceolioscopic (laparoscopic) surgery. We then fit the testosterone concentrations to lognormal distributions and identified the concentration below which individuals are more likely female, and above which individuals are more likely male. Using a parametric bootstrapping procedure, we estimated a 0.01–0.04% misidentification rate for naïve testosterone samples, and a 1.26–1.39% misidentification rate for challenged (post-FSH injection) testosterone samples. Quantification of plasma testosterone concentration from small volume (0.1 mL) blood samples appears to be a viable, highly accurate method to identify sex of 0–3 month old hatchlings and could be a valuable tool for conservation measures and investigation of trends and variation in sex ratios for in situ wild nests.

## Introduction

Chelonians (Order Testudines) are one of the most imperiled groups of vertebrates, with an estimated 56.3% of recognized species at “threatened” or higher risk of extinction by the International Union for Conservation of Nature’s Red List (IUCN)^1^. One aspect of their biology that may render turtles particularly vulnerable to climate change is temperature-dependent sex determination (TSD), which has been documented or is strongly suspected to occur in 262 of the 356 turtle species^2^. In species with TSD, sex determination occurs during a critical time of the egg’s incubation period when the environmental temperature initiates a biochemical cascade resulting in the differentiation of the gonads into ovaries or testes^3^. In sexually reproducing species with TSD, hatchling sex ratios can be severely skewed, in contrast to those species with genetic sex determination that have a primary sex ratio of 1:1^4^. If the population’s sex ratio is sufficiently skewed over time, the ability of the population to persist may be compromised due to demographic collapse^5,6^. It is for this reason that identifying the sex of hatchling turtles is of vital importance for conservation efforts as well as for research into sex-based biological processes such as differential movement, growth, survival, and population dynamics, and how these processes may be linked to a changing climate.

Identifying hatchling sex for turtle species with TSD can be challenging because individuals can remain sexually monomorphic for several years after hatching^7–9^. Available methods for identifying hatchling sex for sexually monomorphic hatchlings vary widely in cost, invasiveness, effectiveness, and generality. Noninvasive methods include morphometric measurements for species with weak sexual dimorphism^10–12^. The minimally invasive method of steroid hormone quantification in blood samples has been long-established for turtles^13^, but is not always successful for distinguishing sex of hatchlings^14^. More accurate methods tend to rely on direct examination of gonads, either via nonlethal surgical methods (e.g., coelioscopic/laparoscopic surgery) or lethal sampling (e.g., histological sectioning of the gonads). Turtle species can vary in their degree of sex differentiation in morphological markers, gonadal development, and biochemical markers at hatching, resulting in the need to validate any method to the individual species of concern.

A species that typifies some of the challenges surrounding sex identification of hatchling turtles is the Mojave desert tortoise (*Gopherus agassizii*), listed as threatened under the U.S. Endangered Species Act in 1990^15^ and considered “critically endangered” with a decreasing population trend by the IUCN^16^. This tortoise appears externally sexually monomorphic until sexual maturity, with some adults remaining externally sexually ambiguous even into late maturity^17^. Correctly identifying the sex of hatchling *G. agassizii* is necessary in studies attempting to investigate population vital rates such as hatchling sex ratio or sex-biased juvenile dispersal, and particularly in regards to projecting resilience to climate change, as skewed population sex ratios of adults have been documented in the wild^18^.

Foundational work on sex identification in juvenile *G. agassizii* was performed by Rostal et al.^19^ who demonstrated that blood plasma testosterone concentration was a successful, minimally invasive method for identifying the sex of 11 months old *G. agassizii* with 98% accuracy that corresponded with identification made by more invasive coelioscopic surgery. Two other studies have considered noninvasive linear morphometric measurements to identify sex of juvenile *G. agassizii*^20,21^. The smallest size that yielded accurate identification was 140 mm straight midline carapace length^21^, or approximately 11–12 years old^22^, but the technique failed for smaller or younger individuals. The researchers cautioned that population-level validation for this method was necessary due to the subtle variations in the relationships among the predictive linear measurements^20,21^. There remains a crucial need for a method that identifies the sex of live hatchlings in their season of hatching with high accuracy and that is suitable for a single in situ encounter with minimal processing time because of the legal and ethical considerations when performing research on federally- and state-protected wild animals.

One possible method that might address this need (minimally invasive, successful for 0–3 months old hatchlings, minimal processing time) and that takes advantage of improvements in hormone quantification techniques since Rostal et al.^19^ is an enzyme-linked immunosorbent assay (ELISA, sometimes termed EIA) to quantify blood plasma testosterone concentration. The radioimmunoassay (RIA) technique requires approximately 0.1–0.2 mL plasma or 0.5 mL whole blood^19^, and this volume is much larger than the 0.1 mL whole blood that would be safe to collect from a 0 day old hatchling averaging 20.8 g body mass in one sampling event^23^. An ELISA requires much lower sample volume than RIA (approximately 0.01–0.05 mL plasma or 0.1 mL whole blood depending on test range and sample concentration) and this volume is appropriate for a single sampling occasion with 0 day old hatchlings. However, it remains unclear whether plasma testosterone concentration is strongly differentiated between female and male *G. agassizii* hatchlings, and recent work indicates that hatchling testosterone levels are only weakly predictive of sex for the congeneric *G. polyphemus*^14^. In some turtle species, injection of follicle-stimulating hormone (FSH) into the coelomic cavity has been demonstrated to temporarily induce increased plasma testosterone in males as soon as 60 min after injection, with a smaller increase in females^24^. Therefore, FSH injections prior to blood sample collection (FSH challenge) may enable more robust sex determination in these species.

We investigated whether blood plasma testosterone concentration was a viable method for identifying the sex of 0–3 months old hatchling *G. agassizii*. For this investigation, our primary research hypotheses were that (1) naïve blood plasma testosterone concentration of 0–3 months old hatchling *G. agassizii* differentiates males from females, and (2) blood plasma testosterone concentration of 0–3 months old hatchling *G. agassizii* challenged with FSH differentiates males from females. We tested these hypotheses with hormone quantification and coelioscopic sex validation in captive hatchlings, and compared testosterone concentration in blood samples from hatchlings from wild *G. agassizii* clutches.

## Results

### Naïve testosterone and sex identification (H_1_)

Regarding our first hypothesis, we observed that among the approaches for which “unknown” sexes were not allowed, the probabilistic method at 50% certainty level or using a continuous approach for the naive samples had comparable, very low rates of misidentification of sex (~ 0.01%; Table 1). The probabilistic method for other certainty levels and the empirical range method for naïve samples also had very low misidentification rates, but all had some number of unknown sex (Table 1). These results supported our first hypothesis, that naïve testosterone concentration successfully identifies sex of hatchling *G. agassizii* (42–53 mm straight midline carapace length, 18–34 g).

**Table 1:** Monte Carlo simulations for identifying sex of hatchling Mojave desert tortoise (*Gopherus agassizii*) using threshold values of testosterone concentrations such that values lower than the threshold are more likely to be female (F) with the indicated level of certainty, while values above the threshold are more likely to be male (M) with the indicated certainty. Simulations that assigned unknown sexes were excluded from the calculation of the probability of misidentification of sex. Asterisk (*) indicates departure from 50:50 expected proportion of assigned F:M sexes in the 20,000 simulations. *FSH* follicle stimulating hormone.

FSH challenge	Level of certainty	Threshold (testosterone pg/mL)	Number of simulations assigned each sex (*n* = 20,000)			Percent (%) misidentified	
			F	M	U	F	M
**Empirical range-based approach**							
Naïve		F ≤ 20.8 < U < 125.4 ≤ M	9610	9399	991	0.00	0.00
Challenged		F ≤ 114.7 < U < 139.1 ≤ M	9810	9915	275	1.11	1.00
**Probabilistic approach**							
Naïve	≥ 99.99%	F ≤ 23.3 < U < 56.5 ≤ M	9788	9982	230	0.00	0.00
	≥ 95.00%	F ≤ 32.7 < U < 43.2 ≤ M	9981	9998	21	0.01	0.00
	≥ 80.00%	F ≤ 35.3 < U < 40.3 ≤ M	9991	10,000	9	0.02	0.01
	> 50.00%	F < 37.7 < M	9993	10,007		0.04	0.01
	Continuous		9998	10,002		0.02	0.04
Challenged	≥ 99.99%	F ≤ 17.5 < U < 423.2 ≤ M	1869*	7268*	10,863	0.03	0.00
	≥ 95.00%	F ≤ 73.2 < U < 219.8 ≤ M	8920*	9185*	1895	0.35	0.37
	≥ 80.00%	F ≤ 99.7 < U < 167.1 ≤ M	9587	9655	758	0.77	0.73
	> 50.00%	F < 129.7 < M	9975	10,025		1.39	1.26
	Continuous		9990	10,010		3.25	3.15

### FSH and sex identification (H_2_)

**Figure 1:**
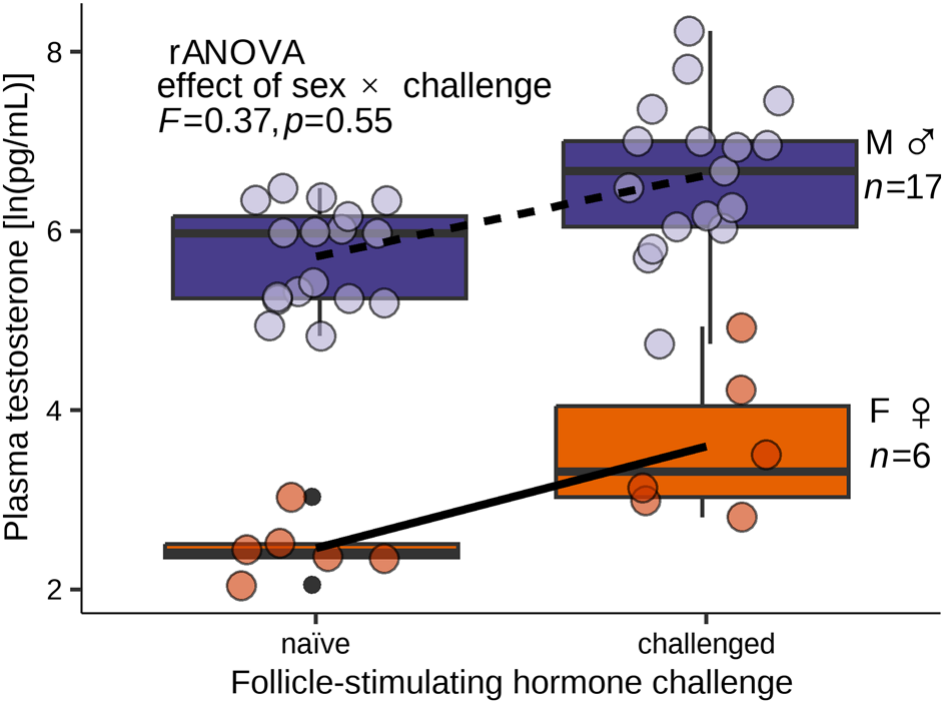
Mean change in blood plasma testosterone concentration from naïve sample to sample collected after follicle-stimulating hormone (FSH) challenge for captive hatchling Mojave desert tortoise (*Gopherus agassizii*; *n* = 23) in Henderson, Nevada, USA in 2019. Sex was validated by coelioscopy at ~ 1.3 year old. Regression lines from a repeated-measures analysis of variance (rANOVA) by sex and challenge status are overlaid on box plots of the log-transformed data. Data points have been horizontally jittered for clarity.

**Figure 2:**
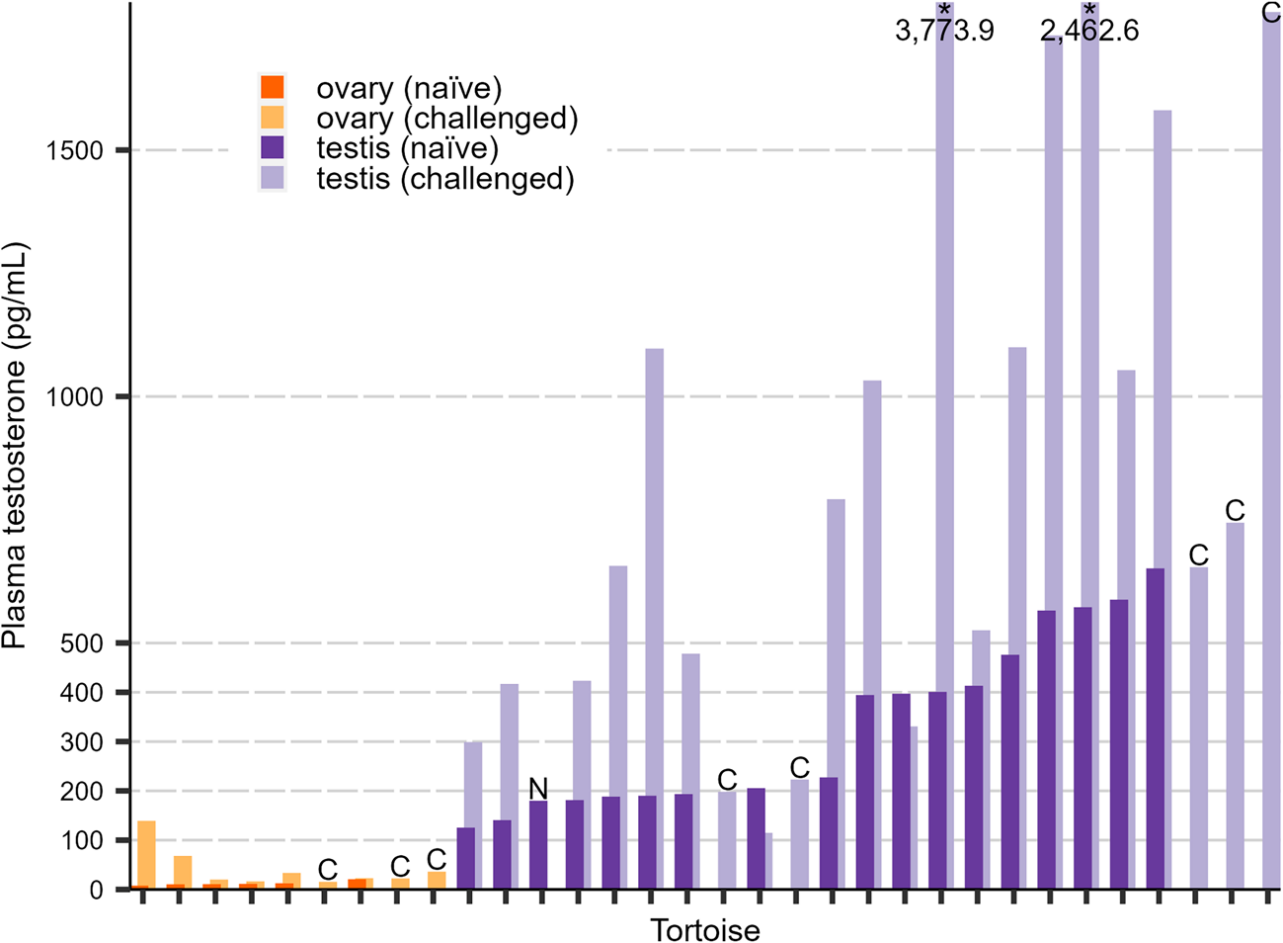
Plasma testosterone concentrations for hatchling (0–3 months old) Mojave desert tortoises (*Gopherus agassizii*) from Clark Co., Nevada, USA before and after follicle-stimulating hormone (FSH) challenge performed in 2019, with sex validated by coelioscopy at ~ 1.3 years old. Data are ordered by naïve concentrations, and naïve/challenged samples are paired by individual. Individuals with only one sample are indicated with a letter above the column (N = naïve only, C = FSH-challenge only). Asterisk (*) indicates column was truncated for visualization and the actual concentrations are written below.

Our model for the effect of sex and FSH challenge on paired pre- and post-challenge samples from 23 individuals failed to show an interaction effect between sex and challenge status (*F* = 0.37, *P*-value = 0.55; Fig. 1). Both females and males increased testosterone concentration in challenged samples over the naïve samples with few exceptions (Figs. 1, 2). We failed to show that FSH differentially elevates male testosterone and concluded that FSH did not induce a mean response to FSH in males that was different from the mean response of females.

Across both the probabilistic and empirical range methods of sex identification, the challenged samples performed poorly compared with the naïve samples, showing much higher proportions of the simulations assigning unknown sex or with higher misidentification rates (Table 1). Unsurprisingly, as the certainty level increased for the probabilistic threshold approach, the misidentification rates decreased and the number of assigned “unknown” sex increased (Table 1). The total proportion of females:males assigned from the 20,000 simulations deviated from 50:50 when using the probabilistic threshold approach on challenged samples at the 95% certainty level (49.3:50.7; *Z*_1_ = 3.55, *P*-value = 0.049) and at the 99% certainty level (20.5:79.5; *Z*_1_ = 3190.2, *P*-value =  < 0.001) (Table 1). Notably, these were the only two sets of simulations that assigned “unknown” sex to more than 1000 replicates.

### Coelioscopy

**Figure 3:**
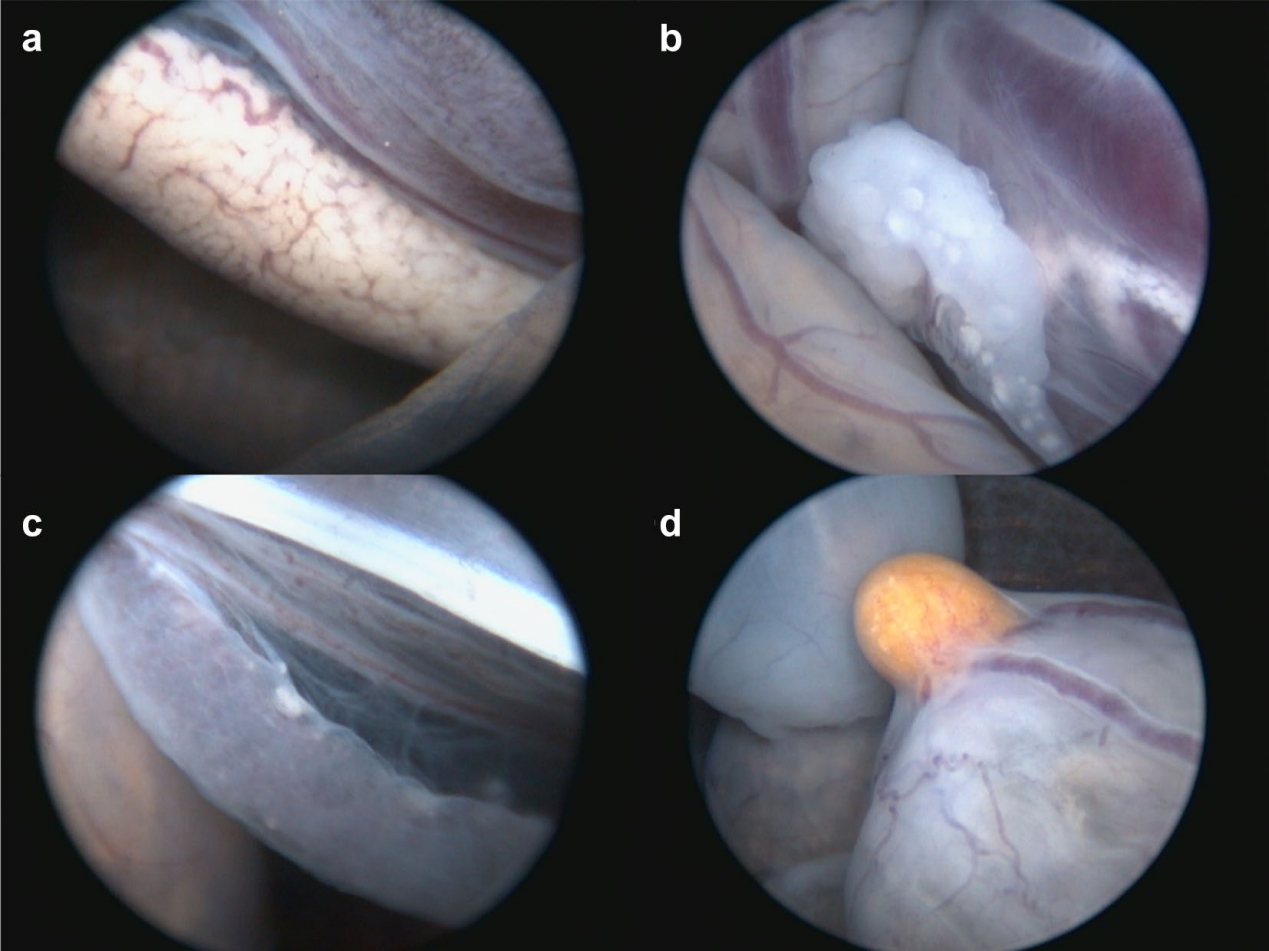
Coelioscopic images of gonads from captive hatchling (0–3 months old) Mojave desert tortoise (*Gopherus agassizii*) performed in Clark Co., NV, USA in 2021. (**a**) Well-differentiated immature testis; (**b**) Well-differentiated immature ovary demonstrating several follicles; (**c**) Poorly differentiated gonad; and (**d**) Small retained yolk sac or Meckel’s diverticulum on the small intestine.

We identified ovaries in nine tortoises from three cohorts and testes in 22 animals from six cohorts. Because we only received origin data at the household level, we considered all individuals collected from the same household as the same “cohort”, thereby representing a conservative estimate of clutch identity. We were unable to accurately sex one individual by ceolioscopy (Fig. 3). Two individuals had retained yolk sacs and we classified them as “abnormal”. We had a single case of bladder perforation that was caused during hemostat or endoscope entry. Given the complexity of repair, time restrictions, and potential for post-operative complications, we elected to euthanize this individual while under anesthesia. We administered naloxone to one individual after it failed to recover from atipamezole, but the tortoise did not respond and was subsequently euthanized. We did not detect any gross anatomical abnormalities during necropsy of any of the euthanized tortoises. All 31 surviving individuals resumed normal activities and exhibited mass gain over the active season subsequent to surgery until entering hibernation in October 2021.

### ELISA validation

**Figure 4:**
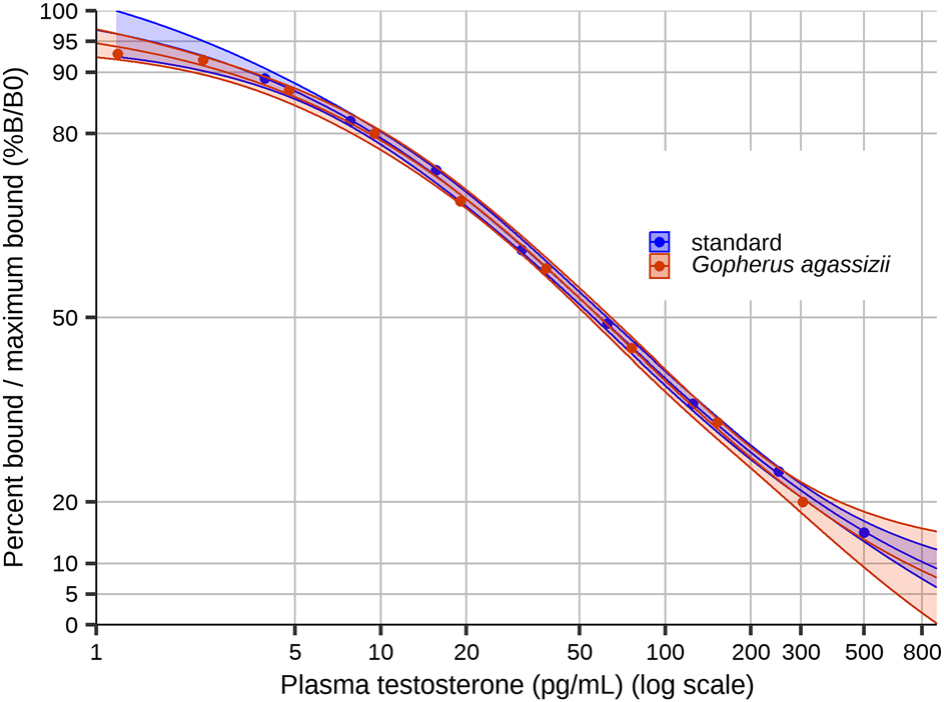
Four-parameter logistic regression curves for the standard dilution series and the pooled sample dilution series, including 95% confidence intervals, for Mojave desert tortoise (*Gopherus agassizii*) blood plasma testosterone concentration determined from enzyme-linked immunosorbent assay. Samples were collected in San Bernardino Co., CA, USA in 2015.

As a prerequisite to testing our two hypotheses, we demonstrated parallelism between our pooled adult female sample and the standard curve as the nested “reduced” model (all four parameters of the logistic regression model) was not different from the alternative “full” model that held slope and inflection point (aka “effective dose 50%”) parameters as different between the standard dilution curve and the pooled sample dilution curve (${\rm X}_{2}^{2}$ = 1.337, *P*-value = 0.213; Fig. 4). Interplate coefficient of variation (CV) was 1.8% and 13.5% (two plates were erroneously run with a more diluted standard, and their CV was calculated separately), and mean intraplate CV was 11.3% (range 3.1–23.0%).

### FSH challenge

We collected naïve and challenged blood samples from 36 captive hatchlings in 2019, of which 33 survived for validation of sex by coelioscopic surgery in 2021. We a priori excluded naïve samples from six individuals due to known vial identification errors made in the laboratory. Female hatchlings had 12.3 pg/mL mean naïve plasma testosterone concentration (range 7.8–20.8 pg/mL, *n* = 6) and 41.6 pg/mL mean challenged plasma testosterone concentration (range 15.6–139.0 pg/mL, *n* = 9), while male hatchlings had 338.4 pg/mL mean naïve plasma testosterone concentration (range 310.9–651.4 pg/mL, *n* = 18) and 975.8 pg/mL mean challenged plasma testosterone concentration (range 114.8–3773.9 pg/mL, *n* = 22) (Table 2). Females and males were separated by 104.6 pg/mL (female maximum: 20.8 pg/mL, male minimum: 125.4 pg/mL) using naïve testosterone concentrations, but overlapped between 114.8 and 139.0 pg/mL using challenged testosterone concentrations (Table 2, Fig. 2). The unambiguous range that separated challenged males from challenged females (i.e., next highest/lowest female/male concentrations outside the range of overlap) was 68.1–198.1 pg/mL (Fig. 2).

**Table 2:** Blood plasma testosterone concentration (pg/mL) from captive (sex confirmed via coelioscopy) or wild (unknown sex) Mojave desert tortoise (*Gopherus agassizii*) hatchlings in southern Nevada, USA in 2017–2019. Included for captive individuals are estimated parameters of fitted lognormal distributions with Kolmogorov–Smirnov goodness-of-fit tests (“K–S statistic”). Samples are from unexposed (“naïve”) individuals and 4 h after injection of follicle-stimulating hormone (FSH; “challenged”). “CI” = confidence interval (2.5–97.5%), “F” = captive female, “M” = captive male, “U(w)” = wild hatchling of unknown sex.

Sex	FSH	*n*	Mean	SD	Median	Min	Max	*μ* (95% CI)	σ (95% CI)	K–S statistic	*P*-value
F	Naïve	6	12.3	4.4	11.2	7.8	20.8	2.46 (2.23–2.70)	0.29 (0.11–0.43)	0.244	0.795
	Challenged	9	41.6	40.0	22.8	15.6	139.0	3.45 (3.01–3.90)	0.68 (0.34–0.95)	0.238	0.606
M	Naïve	18	338.4	176.8	310.9	125.4	651.4	5.69 (5.44–5.94)	0.53 (0.35–0.69)	0.207	0.376
	Challenged	22	975.8	867.8	700.3	114.8	3773.9	6.54 (6.20–6.90)	0.85 (0.58–1.08)	0.0900	0.987
U(w)	Naïve	48	148.0	268.1	27.1	7.1	1351.7				
	Challenged	29	355.5	481.5	102.1	13.6	2092.1				

We collected naïve blood samples from 48 wild hatchlings from 2017 to 2018, and FSH-challenged samples from 29 wild hatchlings in 2019. These wild hatchlings had 20.8 g mean mass (range 16.6–28.9 g), and 43.8 mm mean straight midline carapace length (range 37.1–49.4 mm). Naïve samples (*n* = 48) had 7.1–1351.7 pg/mL range (Table 2). FSH-challenged samples (*n* = 29) had 13.6–2092.1 pg/mL range (Table 2). We observed wild hatchlings with concentrations within the range that separated naïve females from naïve males (*n* = 23; 47.9%), one wild hatchling with a concentration within the range of overlap of FSH-challenged males and females (*n* = 1; 3.4%), and wild hatchlings within the unambiguous range between FSH-challenged males and females (*n* = 9; 31.0%) (Fig. 2).

### Distribution fitting

**Figure 5:**
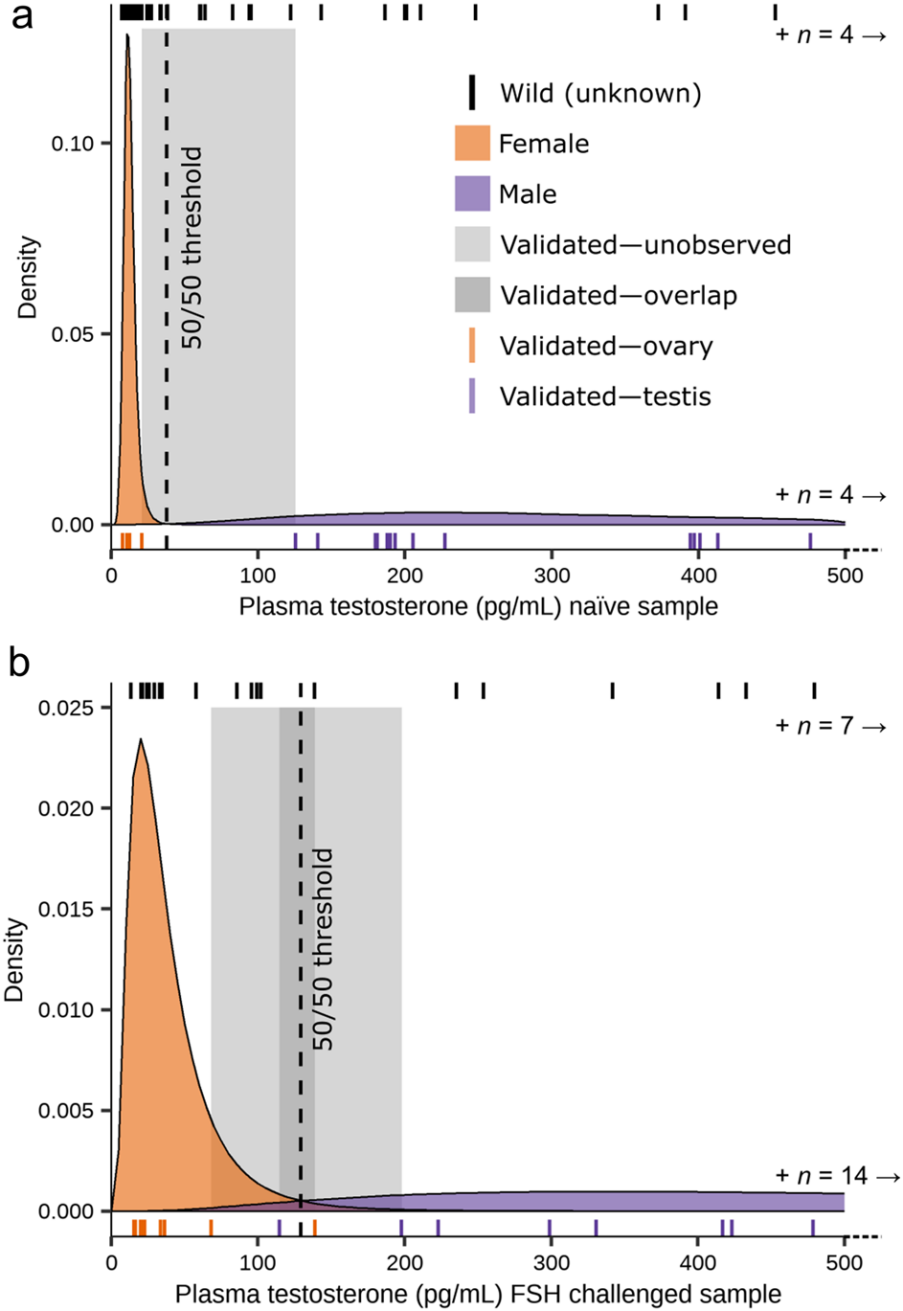
Fitted probability density functions for blood plasma testosterone concentrations of captive hatchling (0–3 months old) Mojave desert tortoise (*Gopherus agassizii*) collected in Clark Co., Nevada, USA in 2019. Sex was confirmed by coelioscopic surgery in 2021. Observed concentrations shown as colored bands beneath density plot. Observed concentrations from wild hatchlings (0 day old) of unknown sex collected in Clark Co., NV, USA 2017–2021 shown as black bands above density plot. Area in gray indicates unobserved concentrations between male and female samples. (**a**) Naïve samples; (**b**) Follicle-stimulating hormone (FSH) challenged samples.

We determined that our sample datasets with known sex followed the lognormal distribution in all cases (Table 2). For almost all datasets, a gamma distribution was an acceptable fit, except that it failed to fit the naïve male samples. Therefore, we chose to use the lognormal distribution for all sample datasets and subsequent analyses (Fig. 5).

## Discussion

In the test of our first hypothesis, we demonstrated that naïve plasma testosterone can be used to identify the sex of hatchlings with very low error. In contrast, we observed overlapping testosterone concentrations of male and female FSH challenged samples, and concluded, for our second hypothesis, that FSH challenge was not effective at identifying the sex of hatchlings. Furthermore, we did not observe a differentiated response between males and females in plasma testosterone concentration following FSH challenge. The coelioscopy protocol for juvenile *G. agassizii* was 97% successful in identifying sex. We validated ELISA for quantifying blood plasma testosterone concentration in *G. agassizii* hatchlings.

Our finding that naïve testosterone distinguishes male from female hatchlings is consistent with previous work by Rostal et al.^19^ on 11 months old *G. agassizii* juveniles. The plasma testosterone concentration we quantified using ELISA in our hatchlings (42–53 mm straight midline carapace length) is on a smaller scale than plasma testosterone quantified by RIA in juveniles (57–90 mm straight carapace length): 7.8–651.4 pg/mL hatchlings versus ~ 19.8–3530 pg/mL juveniles, although for both age groups a clear break between females and males was observed. This pattern indicates that male plasma testosterone concentration continues to increase with increasing size, while female plasma testosterone concentration also does so but at a much slower rate. It is perhaps unsurprising that *G. agassizii* hatchlings show such differentiated plasma testosterone in comparison to *G. polyphemus* that are only weakly differentiated at hatching but become much more so at 4–6 months old; lab-reared individuals are completely separable at 4 years old^14^. While they are congeners, it is estimated that these species diverged 17 mya^25^. Although the developmental stages of the gonads are similar during embryonic development between these species^26,27^, species-specific physiological constraints from different habitats and selection pressures may explain the different patterns of testosterone concentration in the sexes; however, an investigation of the evolutionary origins of this trait difference is beyond the scope of this paper.

We identified the threshold of 37.7 pg/mL naïve testosterone that can be used to identify the sex of hatchling *G. agassizii* with very low error. Although we observed a large gap between the confirmed female with the highest testosterone and confirmed male with the lowest testosterone, our sample size was relatively small, representing six females from two cohorts and 18 males from five cohorts. However, our model did indicate some small area of overlap of the distributions of the testosterone concentrations for males and females. Indeed, we found that a substantial fraction of wild hatchling testosterone concentrations fell within that gap observed between the captive male and female hatchlings. It may be that even the short time in captivity from hatching to sampling experienced by the captive hatchlings (up to 3 months) may have been sufficient to further differentiate testosterone production in males from females. Another possible confounding factor could be maternal effects on offspring testosterone levels. These maternal effects might be a result of the maternal steroid hormones deposited in yolk, as has been observed in painted turtles (*Chrysemys picta*)^28^ (but see Ref.^29^). For these reasons, some of the wild hatchlings that fall within the observed gap between captive males and females may not be as easily differentiable based on ELISA of naïve testosterone; however, our probabilistic method allows for the calculation of the probability of misidentification of sex even for samples within that gap. We concluded that a single blood sampling collection occasion is appropriate and effective to identify sex of catch-and-release wild hatchlings using naïve testosterone quantification.

In this study, we fitted parametric distributions for male and female hatchling testosterone concentrations, and used these distributions to infer thresholds for sex identification. We do not have information on whether the parameters of these fitted distributions might shift between populations such that the optimal threshold is different elsewhere. Despite this source of uncertainty, our parameterized distributions offer a tool for research and conservation efforts that is helpful in circumstances when validation by coelioscopy or other more accurate sex identification methods are not possible, such as when identifying hatchling sex ratios of wild nests in the field. For some applications, a definite identification using the empirical range method is likely to be required; for example, when determining treatment groups for an experimental study or assigning individuals to headstarting cohorts. For other applications, the probabilistic method can yield data that enable improved inference, particularly for in situ field studies and Bayesian modeling approaches using these estimates to inform prior distributions. This probabilistic method based on sex-specific parameterized distributions of plasma steroid hormones could also prove useful for improved inference of sex identification in other turtle species that exhibit less-clear breaks in concentration ranges between males and females.

Interestingly, we failed to find support for our hypothesis that FSH better differentiates male from female hatchlings when compared to naïve testosterone alone. Several previous studies found support for the effectiveness of FSH in raising plasma testosterone concentration in male and not female hatchlings or juveniles. We propose two explanations for our contradictory findings. First, some previous studies that have been successful in using FSH to identify sex used a presence-absence approach: challenged males produced measurable testosterone, while challenged females did not produce any detectable testosterone^24,30^. In contrast, Rhen et al.^31^ tested FSH on juvenile red eared sliders (*Trachemys scripta*), a species in which both male and female hatchlings exhibit detectable levels of naïve testosterone. That study found no effect of FSH on log-transformed testosterone levels in either sex. Recently, however, Loope et al.^14^ found that FSH differentially elevated testosterone in male versus female *G. polyphemus* hatchling using a permutation *t*-test on both absolute and percent change pre- to post-challenge. We chose to compare the relative change in log-transformed testosterone concentration between males and females in this study. By comparing the relative change, we can determine whether both sexes are responding similarly to FSH, i.e., increasing testosterone production at the same rate. Comparing the absolute change between the sexes might, however, be more effective in other species with undetectable levels of testosterone in one or both sexes. We provide the results of a permutation *t*-test on the absolute and percent differences between pre- and post-challenge samples by sex for direct comparison with Loope et al.^14^ in Supplementary Table S1.

Our second explanation for our findings regarding FSH considers our method of injection. We did not have sufficient study animals to determine the lowest effective dose of FSH prior to our experiment, and so we chose to use the lowest effective dose of FSH demonstrated for *G. polyphemus* juveniles. Our dose (0.01 mL) may have been too conservative for *G. agassizii* hatchlings, but we did see increases in testosterone post-challenge for most individuals, both male and female. In addition, we introduced FSH to the hatchling by intracoelomic injection, which is a “blind” injection location that requires needle placement inside the body cavity but away from the organs. If the needle were placed outside the coelomic sac (i.e., between the shell and the coelomic membrane), then the FSH may not have been absorbed in time to induce the expected response of elevated testosterone production. Regardless, the accuracy of sexing based on naïve testosterone obviates the need for the more invasive FSH challenge to resolve sexes in young *G. agassizii*.

Coelioscopy has proven to be a safe and accurate method for identifying sex in a large number of chelonians of different species^32^. Previous studies have demonstrated that injectable anesthetic agents alone may be insufficient and that the addition of local lidocaine improves anesthesia overall^33^. A major advantage of coelioscopy over laboratory-based methods is the ability to directly examine the reproductive tract and identify abnormalities^33,34^. The ability to identify and exclude abnormal animals is critically important in conservation efforts where release of such animals may result in individual suffering (e.g., retained yolk sac leading to omphalitis; abnormal reproductive tract leading to dystocia) or introduce deleterious traits to the free ranging population (e.g., abnormal reproductive tract leading to infertility or reduced fecundity).

The disadvantages of surgical coelioscopy are associated with the risks of anesthesia, surgical trauma (e.g., bladder rupture), and an inability to differentiate between very immature gonads in some species. Such risks are generally very low, but are greatest when a surgeon is inexperienced with the technique or a particular species. There are certainly species-specific considerations regarding safety and gonadal development. The smaller the individual, the greater the anesthetic and surgical challenges. In addition, the stage at which gonads can be reliably identified is consistent within a species, but can be variable between species. For example, the Chinese box turtle (*Cuora flavomarginata*) exhibits obvious gonadal dimorphism at hatching and can be reliably sexed as soon as the yolk sac has been absorbed at about four months and 35 g bodyweight; however, the gonads of radiated tortoises (*Astrochelys radiata*) become difficult to differentiate below about nine months or 100 g body weight^32,33^. To mitigate such risks, it is therefore wise to start with the largest animals in the cohort, and progress to the smaller animals until safety margins or certainty of gonadal identification decrease. In this study, all animals were approximately 1.5 year old and delaying coelioscopy until 2 + years old could decrease the anesthetic and surgical risks, and increase the chances of accurately identifying sex from 97 to 100%.

Although the plasma volume required for the ELISA is much lower than for a radioimmunoassay, a minimum blood draw volume of 0.1 mL may be required for successful completion of the assay. As a caveat, we note that 0.1 mL is the maximum safe blood volume that can be drawn from a 20 g hatchling *G. agassizii*^23^, thus the blood draw volume is necessarily adjusted to the guideline of 0.5 mL/100 g maximum for smaller hatchlings. We found that after sample processing, freezing, and thawing, 0.03 mL plasma was usually sufficient for running the assay in duplicate and yielding results within the 20–80% ratio of bound to total tracer (B/B0 range). The 0.1 mL total blood volume also provided sufficient remaining plasma for an additional sample extraction if another run had been necessary, either due to very low (female) or very high (male) testosterone concentration, requiring a different dilution on the subsequent run for better precision of the determination of testosterone concentration.

Several promising minimally-invasive methods are being developed that might offer higher accuracy and greater certainty in sex identification than the quantification of blood steroid hormone concentration by ELISA, but have yet to be explored for this and many other species of turtle. For example, the presence of anti-Müllerian hormone detected by Western Blot from a small blood sample was successfully used to identify sex of hatchling *T. scripta* and loggerhead turtles (*Caretta caretta*)^35^ (but see Ref.^14^). Sequencing of the DNA methylome of red blood cells and subsequent modeling of 24 loci yielded 100% accuracy in identifying sex of hatchling American alligators (*Alligator mississipiensis*)^36^, but no clear sex-specific diagnostic signal from DNA methylation in tail tissue was found in hatchling *C. picta*^37^ nor in skin tissue in juvenile green sea turtles (*Chelonia mydas*)^38^. Differential gene expression was identified in the transcriptomes of brain and gonadal tissue from male and female hatchling *C. caretta*, suggesting application to sex identification of hatchlings^39^, and blood transcriptomes have been assembled for multiple sea turtle species^40^ and *C. picta*^41^, but it is yet unclear whether differential gene expression in blood samples could be used to identify sex in hatchling turtles with TSD.

The quantification of blood plasma testosterone concentration by ELISA is an effective and accurate method for identifying the sex of hatchling *G. agassizii* in their season of hatching. Sufficient blood volume can be drawn safely from an animal on a single sampling occasion in situ, with the only major on-site resource requirement being centrifugation to separate the blood plasma within four hours of collection, and dry ice or ultracold freezer for storage until the samples can be assayed. We provide our validated empirical ranges for distinguishing male from female hatchlings. Our parameterized distributions of the probability of testosterone concentration being from a male or female allow for a less conservative, probabilistic method to assign sex even within the unobserved range of concentration between the highest concentration female and the lowest concentration male.

## Methods

### Ethics declarations

We performed this research with permissions from Nevada Department of Wildlife SCP #40292, University of Nevada, Reno Institutional Animal Care and Use Committee Protocol #20081068, and U.S. Fish and Wildlife Service Recovery Permit TE50049D-2. We performed all methods in accordance with the guidelines and regulations of the University of Nevada, Reno Institutional Animal Care and Use Committee. Authors complied with the ARRIVE guidelines.

### Captive study animals

Candidate captive juvenile *G. agassizii* (< 100 mm straight midline carapace length) under the care of the U.S. Geological Survey, Western Ecological Research Center (Henderson, NV, USA) were evaluated for apparent age. We selected individuals that we estimated were 0–3 months old between August–November 2019 based on having zero scute annuli surrounding the natal scute, as well as size and weight that were consistent with wild *G. agassizii* hatched during those months. These captive individuals originated from a broader pool of juveniles that were voluntarily surrendered from households in the Las Vegas valley (NV, USA) during this time period, and so parentage and exact date of hatching were unknown. Captive individuals remained under the care of the U.S. Geological Survey during the study period (2019–2021). We attempted to collect both naïve and FSH-challenged samples from these candidates.

### Wild study animals

Wild hatchling *G. agassizii* were sourced from wild nests with known dates of hatching in 2017–2021. To find nests, we tracked wild gravid female *G. agassizii* using radio telemetry, then located and caged their nests in situ to prevent depredation. We monitored nests until hatching. Once hatched, we collected a blood sample from every wild live individual for naïve testosterone in 2017–2021, excluding 2019. No additional (i.e., FSH-challenged) samples were collected from these individuals due to blood volume constraints. In 2019, we challenged hatchlings with FSH, then collected a challenged sample, and no additional (i.e., naïve) samples were collected from these individuals due to blood volume constraints.

### Blood sample collection

Upon each sampling occasion (including both naïve and FSH-challenged samples), we collected up to 0.1 mL blood from the subcarapacial cervical plexus of hatchlings^42^. For wild tortoise sample collection in 2019 and all captive tortoise sample collection, we manually coated 27-G, 5/8-in needles and 1-mL syringes with liquid sodium heparin (10,000 units) prior to collection to prevent the sample from clotting^43,44^. After drawing 0.1 mL heparin into the syringe, we completely depressed the plunger, then drew air and cleared the syringe three times. This technique decreases, but does not eliminate, a dilution effect from the liquid heparin coating^43,45^. We judged the use of liquid heparin to be necessary because it substantially increased the success of collecting a sample. We did not coat needles in heparin for wild tortoise samples collected in 2017, 2018, and 2021. Once drawn, we transferred the sample to 70-μL heparinized capillary tubes that we placed on wet ice. Within 4 h of sample collection, we centrifuged capillary tubes at 1200 rpm for 3 min, recorded the hematocrit concentrations, and transferred the plasma portion of each tube into separate cryovials for storage at – 70 °C until laboratory analysis.

### FSH challenge

Upon intake of captive hatchlings, we collected a blood sample for the determination of naïve plasma testosterone concentration. All sampled captive hatchlings remained overnight for observation before we returned them to the care of the U.S. Geological Survey. If we failed to collect a naïve blood sample after three draws with a needle, we made a second collection attempt after at least seven days had elapsed to allow time for healing at the sample collection site.

At least 7 days after the collection of the naïve blood sample, or upon the emergence of a wild hatchling from the nest in 2019, we injected 0.1 mL of porcine-FSH solution (0.1 units/1 mL sterile saline; MP Biomedicals LLC, Irvine, CA, USA) for a total dose of 0.01 units p-FSH into the coelomic cavity following the method of Lance et al.^24^. This dosage was chosen because it was the lowest tested dose that resulted in the elevation of male testosterone in congeneric *G. polyphemus* hatchlings^14^. Four hours after FSH challenge, we drew a challenged blood sample. All challenged captive hatchlings were kept overnight for observation before being returned to the care of the U.S. Geological Survey. Individuals were only challenged with p-FSH once; if we failed to collect a blood sample after three draws with a needle, we removed the individual from our study.

### Coelioscopy

When the captive hatchlings that we challenged with FSH were approximately 1.3–1.6 year old in April 2021 (*n* = 33), we definitively sexed them using endoscopic surgery; i.e., visual inspection of the gonads from within the coelom^19,32,33^). We fasted tortoises for 24 h prior to surgery to prevent regurgitation during anesthesia. We soaked all animals in shallow aged tap water for 30 min at least 12 h before surgery to encourage urination and reduce bladder size.

On the day of surgery, we made all intramuscular and subcutaneous injections with 31-G, 8-mm needles and 0.3-mL syringes, and sterilized equipment in CIDEX OPA Solution (Advanced Sterilization Products, Irvine, CA USA). Following aseptic preparation of the left pectoral limb with 70% isopropyl alcohol, we injected a general anesthetic mixture of ketamine (20 mg/kg), hydromorphone (0.5 mg/kg), and dexmedetomidine (0.075 mg/kg) into the left pectoralis major muscle. After completing the procedure on five individuals, we determined that the anesthetic doses could be reduced by 17% for all subsequent individuals (*n* = 29). Following administration of anesthetic drugs, we placed animals in an incubator maintained at 29 °C for 30 min.

Following anesthetic induction, and after confirming no response to a toe pinch stimulus (i.e., adequate depth of anesthesia), we prepared each individual for surgery by positioning in right lateral recumbency on a soft, clean, rolled towel. We secured the left pelvic limb extended caudad using medical tape. We aseptically prepared the left prefemoral fossa by alternating three times with 1% chlorhexidine gluconate (Hibiclens, Mölnlycke Health Care, Norcross, GA, USA) diluted with sterile water, and 90% isopropyl alcohol. We infiltrated subcutaneously lidocaine (2% Lidocaine HCl, Hospira Inc, Lake Forest, IL, USA) diluted 1:10 with sterile water, into the left prefemoral fossa at a maximum dose of 4 mg/kg to provide local analgesia. After waiting five minutes for the lidocaine to take effect, we made a 2–3-mm craniocaudal skin incision in the center of the prefemoral fossa with a no. 15 scalpel blade. We directed small, straight mosquito hemostats through the incision and aiming craniad, penetrating the coelomic aponeurosis. We replaced the hemostats with a 1.9-mm 30° telescope with integrated sheath (67030 BA, Karl Storz Veterinary Endoscope-America, Goleta, CA, USA) in the coelom. We achieved coelomic insufflation by injecting 1–3 mL of sterile saline containing 1 mg/mL cefazolin (1-g vial Cefazolin, Apotex Corp., Weston, FL, USA added to 1-L bag of sterile saline).

We directed the telescope caudodorsad to inspect the gonads. We identified the gonads as testis, ovary, or undifferentiated, and their gross appearance as normal or abnormal. We removed the telescope once we identified the gonads and recorded images, or after 10 min, whichever occurred first. We permitted any excess insufflation fluid to drain passively prior to routine skin closure using a single, absorbable, antibacterial suture (5/0 Monocryl-Plus with a P3 3/8 circle needle, Ethicon Route 22 West, Somerville, NJ, USA).

After carefully removing all tape, and again following aseptic precautions, we reversed anesthesia by the intramuscular administration of atipamezole (0.5 mg/kg) into the right pectoralis major. We returned each individual to the incubator for 40 min to allow time for anesthetic recovery. Following aseptic precautions, we administered meloxicam (Metacam 5 mg/mL, Boehringer Ingelheim Vetmedica, Inc, Pet Division, St. Joseph, MO, USA), a nonsteroidal anti-inflammatory, subcutaneously (0.2 mg/kg) into the left forelimb to provide additional post-operative analgesia. Then, we again returned individuals to the incubator where they were monitored until resumption of normal activity. If recovery did not occur within 30 min of the atipamezole administration, we gave naloxone (Naloxone HCl 0.4 mg/mL, Hospira Inc, Lake Forest, IL, USA) at 0.05 mg/kg intramuscularly to reverse the effects of the hydromorphone. If an individual experienced a serious adverse event while anesthetized, or failed to recover following the administration of naloxone, we performed euthanasia by pithing after first ensuring the animal was completely insensitive.

We housed tortoises overnight at 27 °C^46^, and reassessed the following morning for attitude, posture, and general condition^47^ before returning them to their outdoor habitat pens. The U.S. Geological Survey continued to maintain the individuals in their outdoor habitat pens, and we performed regular post-surgical examinations over the following six months to record mass, monitor wound healing, and assess behavior and activity level.

### Enzyme-linked immunosorbent assay (ELISA)

We performed all testosterone assays using the commercially-available Cayman Chemical Testosterone ELISA Kit (No. 582701, Ann Arbor, MI, USA), with a reported range of 3.9–500 pg/mL. To extract the free, unbound portion of testosterone from the blood plasma, we pipetted 0.01–0.045 mL plasma into a glass test tube. We then added 1.0 mL diethyl ether, vortexed the tube for 20 s, and then held the tube on dry ice for 20 s. We decanted the organic layer into a clean test tube and dried the sample overnight in a fume hood. We re-eluted the sample with 0.11 mL of ELISA Kit buffer, and then assayed the sample in duplicate following manufacturer’s instructions. If sample volume allowed, we reran samples with a high coefficient of variation (> 20% CV) or reran samples with a different dilution factor if sample concentration was outside 20–80% of the ratio of bound to total tracer (B/B_0_).

We began our validation of this ELISA Kit for *G. agassizii* by developing a pooled sample of wild adult female *G. agassizii* blood plasma samples (*n* = 20) that were collected by the U.S. Geological Survey in 2007^48^ and stored at − 70 °C. As we did not have available known-sex hatchling or juvenile blood plasma samples, we selected these samples because adult female *G. agassizii* have lower testosterone concentrations than males^49^, therefore a pooled sample likely yields values similar to the range of our hatchling samples of unknown sex after fewer dilutions than a pooled male sample^19^. We used a twofold dilution series of the pooled sample to run in parallel with the dilution series of the kit standard. We assessed parallelism first by performing four-parameter logistic regressions to model the two curves with the “drc” package^50^. We then compared a model with both slope (at the inflection point) and the inflection point (aka “effective dose 50%”) as different between the two sets of dilutions (assuming the upper and lower limits of the curves were the same), a model with only slope different, a model with only the inflection value different, and the nested model with all four parameters (upper limit, slope, inflection point, and lower limit) the same using the likelihood ratio test with the “lmtest” package^51^. We considered extraction efficiency to be equal to that determined using this same kit for *G. polyphemus* blood plasma (94.1% ± 16% SD)^14^. Cross-reactivity and sensitivity were reported in the kit manual.

### Statistical analyses

We performed all analyses in R version 4.1.1^52^. We set the Type I error rate (*ɑ*) at 0.05 for all statistical tests.

#### Distribution fitting

A competitive ELISA uses the ratio of bound to total tracer (%B/B_0_) to calculate testosterone concentration of the sample, and a conservative approach is to reject samples outside the linear portion of the standard curve, estimated at 20–80% B/B_0_. However, for applications that allow somewhat greater error around the estimates, 10–90% B/B_0_ is acceptable, as 90% B/B_0_ is considered the lower limit of detection. We observed *n* = 1 sample below 10% (9.91%) and *n* = 2 samples above 90% (91.03% and 92.44%). Because we were willing to accept somewhat greater error in the lower tail of the female distribution and the upper tail of our male distribution, we decided to use all sample data for distribution fitting. We fit each subset of data defined by challenge status and sex (e.g., “naïve female”, “FSH-challenged male”, etc.) separately to a lognormal, gamma, or Weibull distribution, and determined the 95% confidence intervals around the parameter estimates using nonparametric bootstrapping with 10,000 iterations with the “fitdistRplus” package^53^. Next, we identified whether the distribution was a good fit using the Kolmogorov–Smirnov test implemented in the “EnvStats” package^54^. We further examined the quantile–quantile plots and expected cumulative density function versus theoretical cumulative density function to confirm whether the selected distribution represented each dataset well.

#### Effect of FSH

We tested whether the mean change from naïve to FSH-challenged samples was different in males versus females by performing a repeated measures ANOVA on paired samples for each confirmed-sex individual. We set sex as a between-subjects factor, while challenge status and the interaction between sex and challenge status were within-subjects factors. The dependent variable, testosterone concentration, was log-transformed to meet the assumption of normality if necessary. We performed Levene’s Test with the “car” package^55^ to test the assumption of homogeneity of variance. We performed the Shapiro–Wilk test of normality on the residuals and plotted the residuals with a quantile–quantile plot to investigate whether they were normally distributed. A significant interaction between sex and challenge status demonstrates a difference in the response to FSH between males and females, indicating that follicle stimulating hormone injection was successful in further differentiating males from females.

#### Identification of sex

We addressed our two hypotheses (naïve and FSH-challenged plasma testosterone concentrations differentiate sex of hatchling *G. agassizii*) by calculating the potential for misidentifying the sex of an individual from testosterone concentration. We compared two methods for assigning sex to unknown-sex hatchlings: the “probabilistic approach” and the “empirical range approach”.

The probabilistic approach estimated the relative probability of being male or female by integrating over the density curves of the fitted male and female lognormal distributions at the sample testosterone concentration using a 0.1 pg/mL interval (T_sample_ − 0.05 pg/mL: T_sample_ + 0.049 pg/mL). The relative probability of being male was the ratio of the integral of the binned male density curve to the sum of the integrals of the binned male and female density curves, while the relative probability of being female was the ratio of the integral of the binned female density curve to the sum of the integrals of the binned male and female density curves. If using certainty thresholds, then we assigned sex as “male”, “female”, or “unknown” based on where the sample concentration fell in relation to the threshold. We used four thresholds: 50%, 80%, 95%, and 99.99% thresholds. For example, if using the 80% certainty threshold, we would assign sex as “male” if the sample concentration was *equal to or higher* than the value at which the relative probability of being male was 80%. If, instead, the sample concentration was *equal to or lower* than the value at which the relative probability of being female was 80%, we would assign sex as “female”. Otherwise, if the sample concentration fell *between* those two values, then we would assign it “unknown” sex. If using a continuous approach, then we assigned sex using a coin toss weighted by the relative probability of being male versus the relative probability of being female (no “unknown”) at the sample concentration.

Our second method, the “empirical range approach”, assigned sex “female” if the sample testosterone concentration was *equal to or below* the highest observed female testosterone concentration, “male” if the sample concentration was *equal to or higher* than the lowest observed male testosterone concentration, and “unknown” if the sample concentration was *between* the highest observed female concentration and lowest observed male concentration. If the male and female concentrations overlapped, then we used the first highest female and first lowest male concentrations outside the range of overlap as the range limits.

We used a Monte Carlo simulation approach to estimate misidentification rates in which we sampled testosterone concentrations for hypothetical known-sex hatchlings (on the basis of the fitted lognormal distributions above) and subsequently applied each of the two methods for assigning sex. For each replicate, we first randomly sampled parameter values for the appropriate lognormal distribution (representing male or female testosterone concentration) from a uniform distribution with upper and lower limits defined by the 95% bootstrapped confidence intervals. We then randomly drew a single testosterone concentration from this lognormal distribution and assigned a sex using the two methods described above. We ran a total of 10,000 replicates for each sex and computed the misidentification rate. “Unknown” assignments were not considered misidentification errors. Finally, we used a *Z* test of proportions to determine whether the observed proportion of assigned females to assigned males from all 20,000 replicates was the expected 50:50. A departure from the expected proportion could signal that the method yielded biased sex assignments.

### Supplementary Information

Supplementary Table S1.

## Data Availability

The datasets generated and analyzed during the current study, and all R scripts used in analysis, are available in the GOAG_hormone_sex_identification repository, https://github.com/mawalden/GOAG_hormone_sex_identification.
