# Supplementary material for: Testosterone identifies hatchling sex for Mojave desert tortoises (Gopherus agassizii)
Source: Sci Rep. 2023 Sep 8;13:14818. doi: 10.1038/s41598-023-41677-2 (PMC10491821; doi:10.1038/s41598-023-41677-2)
Supplement: Supplementary file 1 — Supplementary Table S1. [file 41598_2023_41677_MOESM1_ESM.pdf]

Testosterone identifies hatchling sex for Mojave desert tortoises (*Gopherus agassizii*)

M. A. Walden[1,2,*], Kevin J. Loope[3,4], Elizabeth A. Hunter[5], Stephen J. Divers[6], Jessica R. Comolli[6,7], Todd C. Esque[8], and Kevin T. Shoemaker[1]

Department of Natural Resources and Environmental Science, University of Nevada—Reno, Reno, NV, USA
[2] Present Address: Department of Fisheries Biology, California State Polytechnic University—Humboldt, Arcata, CA, United States
[3] Department of Fish and Wildlife Conservation, Virginia Tech, Blacksburg, VA, USA
[4] Department of Biology, Georgia Southern University, Statesboro, GA, USA
[5] U.S. Geological Survey, Virginia Cooperative Fish and Wildlife Research Unit, Department of Fish and Wildlife Conservation, Virginia Tech, Blacksburg, VA, USA
[6] Department of Small Animal Medicine and Surgery, College of Veterinary Medicine, University of Georgia, Athens, GA, USA
[7] Present Address: Department of Veterinary Services, Miami Seaquarium, Key Biscayne, FL, USA
[8] U.S. Geological Survey, Western Ecological Research Center, Boulder City, NV, USA
[*] mar.walden@gmail.com

Supplementary Information

Supplementary Table S1. Permutation *t*-test for paired pre- and post-follicle-stimulating hormone (FSH) challenge blood plasma samples from captive hatchling (0–3 mo old) Mojave desert tortoises (*Gopherus agassizii*) collected in Clark Co., Nevada, USA in 2019. Sex was confirmed by ceolioscopy in 2021. Samples are from unexposed individuals and 4 hr after injection of FSH.

**Sex**	***n***	**Average absolute difference pre- to post-FSH challenge**	**Average percent difference pre- to post-FSH challenge**
Female	6	37.68 pg/mL	422.95%
Male	17	703.45 pg/mL	197.11%

**Permutation *t*-test**	**95% Confidence Interval**	***P*-value**
Absolute difference	–1,205.12–122.30 pg/mL	< 0.001
Percent difference	–5.00–585.92 pg/mL	0.497
